# Considering the Technological and Sensory Needs of Patients With Cognitive Impairment in the Era of Telehealth

**DOI:** 10.1093/geroni/igab046.326

**Published:** 2021-12-17

**Authors:** Esther Oh, Julie Yi, Corrine Pittman, Carrie Price, Carrie Nieman

**Affiliations:** 1 Johns Hopkins University School of Medicine, Baltimore, Maryland, United States; 2 Johns Hopkins University, Baltimore, Maryland, United States; 3 Howard University College of Medicine, Washington, District of Columbia, United States; 4 Towson University, Towson, Maryland, United States

## Abstract

During the COVID-19 pandemic, telehealth has become an important means of delivering memory care. Telehealth that is responsive to the technological abilities and preferences as well as the sensory needs of persons living with dementia is critical to advancing access to care. We conducted a systematic review to investigate the use of telehealth among older adults with cognitive impairment. The search yielded 3,551 titles and abstracts that led to 17 full-text articles. Studies showed that telehealth can be used for routine care, cognitive assessment and telerehabilitation with good efficacy and satisfaction. Three studies investigated telemedicine delivery in the home and 16/17 studies relied on support staff and care partners to navigate technology. No studies reported adaptations to account for sensory impairments and 5/17 studies excluded individuals with sensory impairments. This talk will review barriers and facilitators totelehealth for older adults with cognitive impairment and adaptations to address sensory needs.

